# 

**Published:** 1891-10-24

**Authors:** 


would have to be preferred to the Extractum Carnis, for it would contain all the nutritive constituents of meat." Again—"I have before stated that in prein the residue, they are

"BOVRIL" contains who know the requirements of the human system and the is identical with what the body requires for recuperation, cede any animal aliment at present known.

paring the Extract of Meat, the Albuminous principles remain
lost to nutrition, and this is certainly a great disadvantage."
the albumen and fibrine in the most perfect possible form, and to
constituents of food, it will be apparent that this albumen and
and that as a perfect form of concentrated nourishment it must

SOLD THROUGHOUT THE UNITED KINGDOM.

### THE HOSPITAL

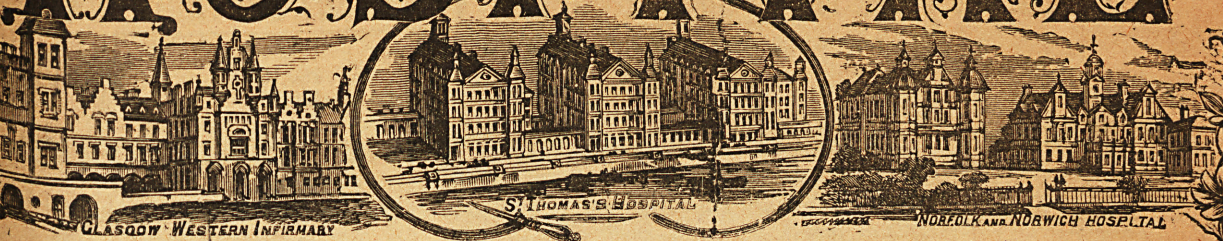

WITH EXTRA NURSING SUPPLEMENT.

No. 265. Vol. XI.] SATURDAY, OCTOBER 24th, 1891. [ONE PENNY.

